# Reconstructed gastric conduit obstruction caused by a bezoar after esophagectomy: a case report

**DOI:** 10.1186/s12893-019-0525-5

**Published:** 2019-06-07

**Authors:** Keiichiro Hatoyama, Yusuke Taniyama, Tadashi Sakurai, Makoto Hikage, Chiaki Sato, Hiroshi Okamoto, Kai Takaya, Yu Onodera, Takashi Kamei

**Affiliations:** Department of Surgery, Tohoku University Graduate School of Medicine/Seiryo-machi, Aoba-ku, Sendai, Miyagi 980-8574 Japan

**Keywords:** Bezoar, Obstruction, Esophageal cancer, Reconstructed gastric conduit, Esophagectomy

## Abstract

**Background:**

Bezoars are rare but may cause gastrointestinal obstruction and ulcers. To the best of our knowledge, only two cases of bezoars in the reconstructed gastric conduit have been reported, but there has been no report on reconstructed gastric conduit obstruction due to bezoars.

**Case presentation:**

A 60-year-old man presented to our clinic with abdominal pain and vomiting that occurred suddenly after dinner. Three years before presentation, he had undergone radical thoracoscopic esophagectomy followed by reconstruction of the gastric conduit through the posterior sternum, for esophageal cancer. Enhanced computed tomography scans showed distension of only the gastric conduit without ischemia and distension of the small intestine. According to our findings, we initially diagnosed the patient with postoperative intestinal obstruction caused by adhesions. After conservative treatment failed, the patient underwent an endoscopic study that showed a bezoar at the pylorus ring. We initially failed to remove the bezoar endoscopically because of its large size; hence, we attempted enzymatic dissolution. Three days after the first endoscopic study, the bezoar was disintegrated using a snare and extracted during a second endoscopy. The patient recovered uneventfully and presented with no complications during the 1-year follow-up interval.

**Conclusion:**

In cases wherein the discharge of materials in the reconstructed gastric conduit is delayed, bezoars should be considered in the differential diagnosis, and an endoscopic study should be performed to verify the cause of obstruction.

## Background

Bezoars are defined as masses of indigestible hard materials that form in the gastrointestinal tract [1] and occur in 0.068–0.4% of the population [2, 3]. Moreover, bezoars may cause gastrointestinal obstruction and ulcers. To the best of our knowledge, there are only two cases of bezoars in the reconstructed gastric conduit [4] but none on reconstructed gastric conduit obstruction caused by bezoars.

Here, we report an extremely rare case of reconstructed gastric conduit obstruction caused by a bezoar after esophagectomy for esophageal cancer.

## Case presentation

A 60-year old man presented to our clinic with abdominal pain and vomiting soon after dinner. Three years prior to this event, he underwent radical thoracoscopic esophagectomy followed by reconstruction of the gastric conduit through the posterior sternum, for esophageal cancer. Past medical history was not significant for any medical condition, such as diabetes, or medication that might cause autonomic disorders.

**Fig. 1:**
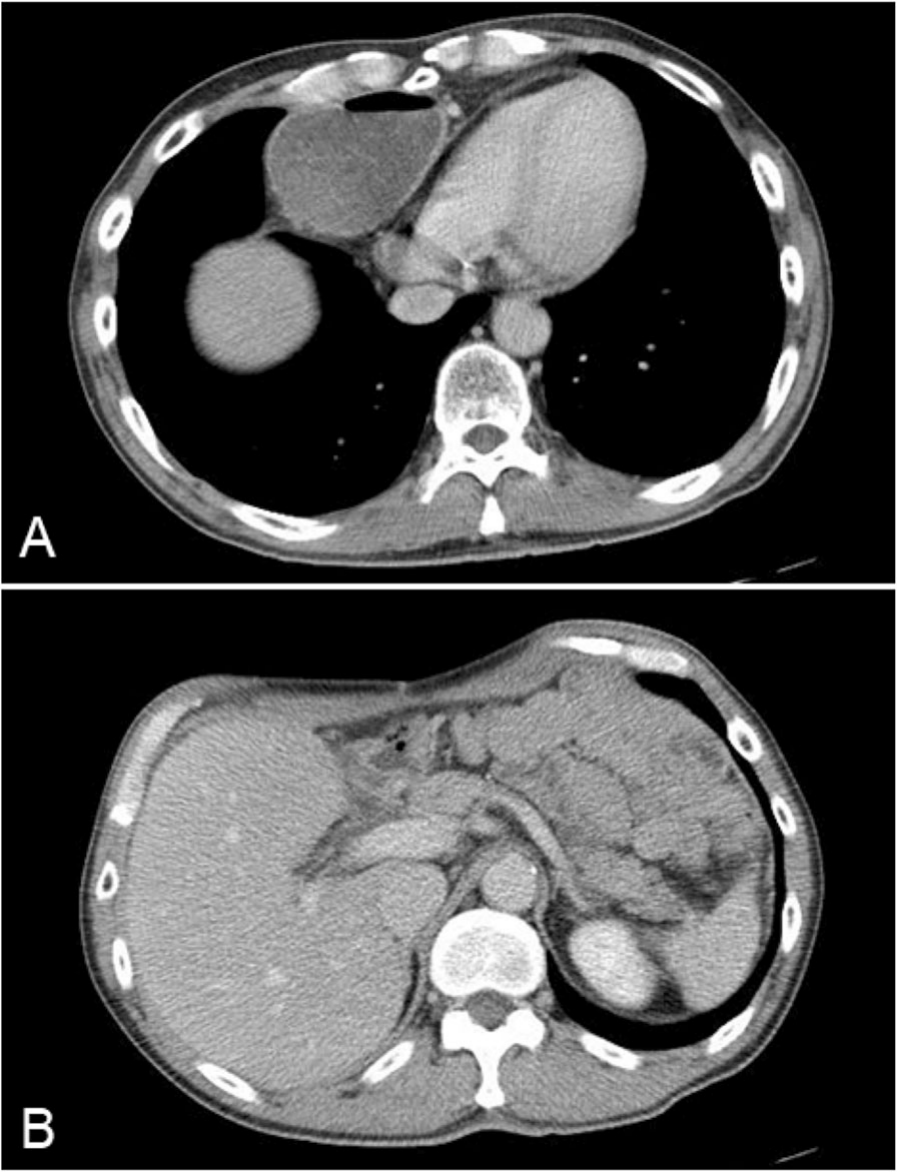
Enhanced computed tomography images. **a**: This image shows distension of the gastric conduit without ischemia. **b**: No obvious structure resembling a bezoar was observed at the pylorus ring, and there was no distension of the small intestine

On admission, his vital signs were normal, and a routine blood test did not indicate any abnormalities. Physical examination with direct palpation revealed right upper abdominal pain without rebound tenderness. Enhanced computed tomography (CT) scans showed distension of only the gastric conduit without ischemia and without distension of the small intestine (Fig. 1). Based on these findings, we initially diagnosed the patient with postoperative upper intestinal obstruction caused by adhesions.

**Fig. 2:**
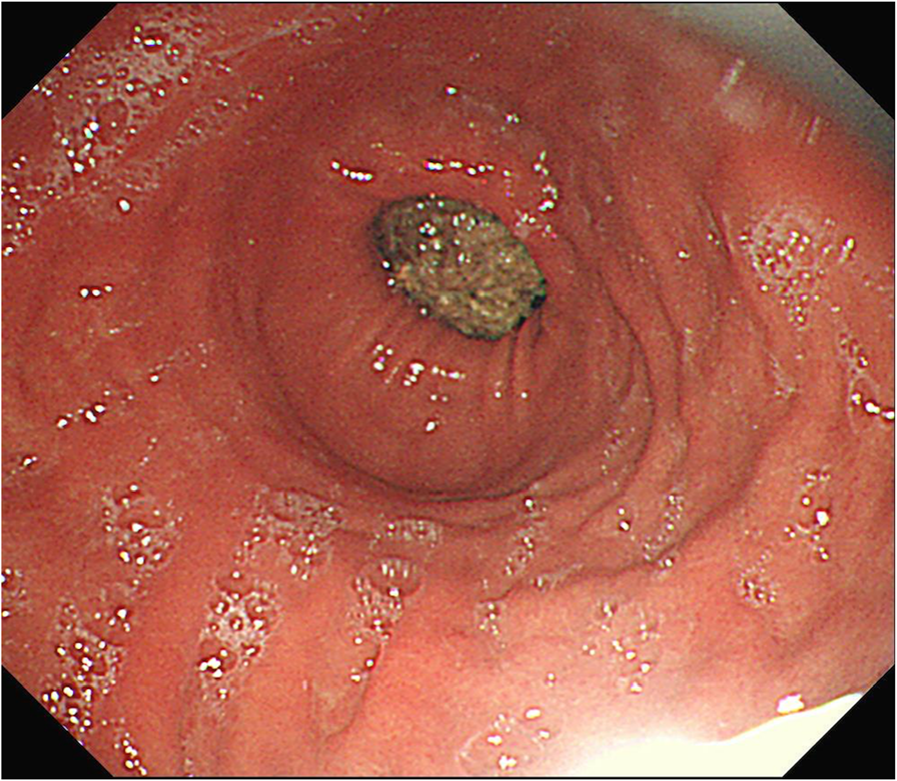
The first endoscopic finding. This image shows the bezoar attached to the pylorus ring

At that time, conservative treatment with nasogastric tube drainage and intravenous fluid supplementation was initiated. The patient’s symptoms gradually subsided and oral feeding was initiated 3 days after the conservative treatment. However, immediately after oral feeding, vomiting recurred. An endoscopic study was then performed for further examination, and a bezoar obstruction at the pylorus ring (Fig. 2) was observed.

**Fig. 3:**
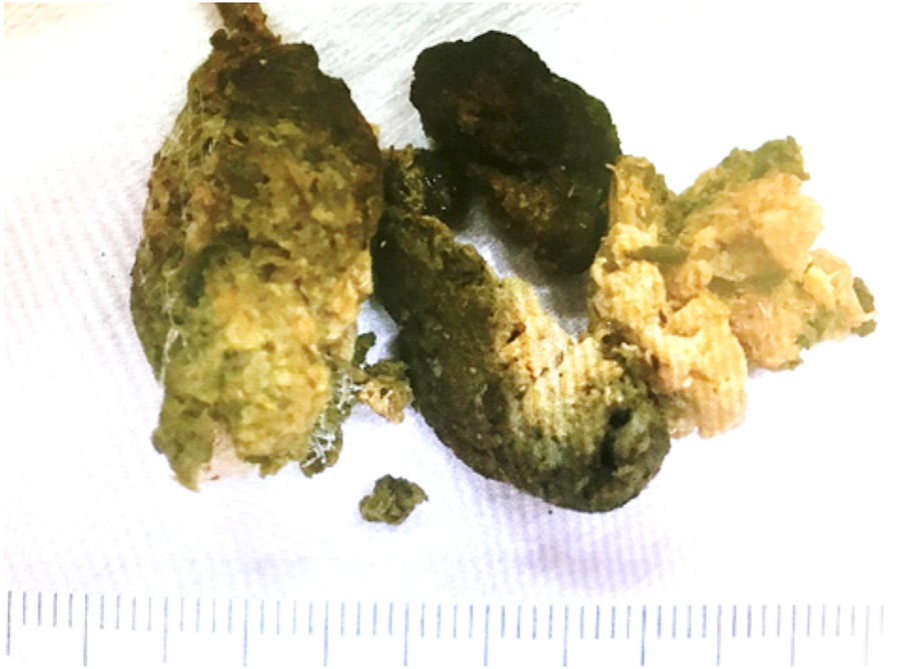
The bezoar. This image shows endoscopically removed fragments of the bezoar

**Fig. 4:**
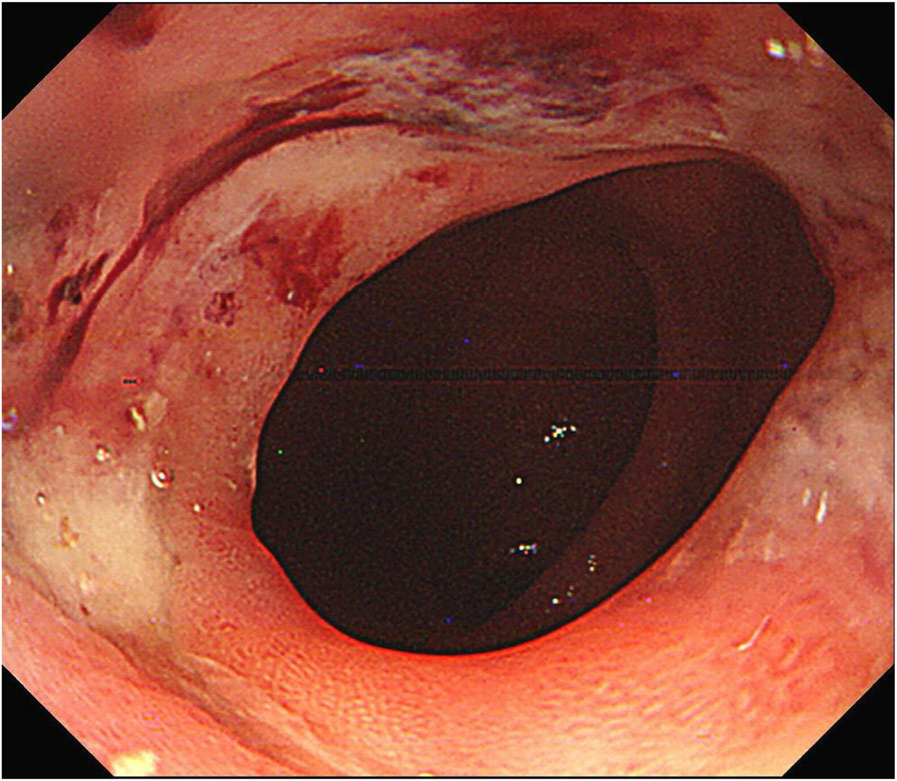
The second endoscopic finding. This image shows an ulcer at the same location as the bezoar

We initially failed to remove the bezoar endoscopically because of its large size; hence, we attempted enzymatic dissolution. Three days after the first endoscopic study, the bezoar was disintegrated using a snare and extracted during a second endoscopy (Fig. 3). The second endoscopic study revealed an ulcer at the same location as the bezoar (Fig. 4); hence, we administered a proton pump inhibitor. The patient recovered uneventfully and presented with no complications during the 1-year follow-up interval.

## Discussion and conclusions

We report an extremely rare case of obstruction of the reconstructed gastric conduit caused by a bezoar, 3 years after esophagectomy for esophageal cancer. The patient was treated successfully and did not experience any complications postoperatively.

Bezoars grow with the ingestion and accumulation of indigestible materials. The major predisposing factors for bezoar formation in the gastric conduit are delayed gastric emptying, inadequate food mixing caused by poor function of the pyloric sphincter due to truncal vagotomy, and poor digestive function of the stomach because of its use for reconstruction in esophagectomy. [4]. In fact, this patient underwent 9 endoscopic examinations before this event, including endoscopic treatment (dilation) for stenosis of the anastomosis. Two of the prior endoscopic examinations revealed large quantities of remnant food in the reconstructed gastric conduit. However, pyloric stenosis was not detected in prior endoscopic examinations.

Other risk factors for bezoar formation include eating habits, such as excessive persimmon consumption; diabetic gastropathy; cerebral infarction; and medications that reduce gastrointestinal motility [5]. The pathogenesis is very complicated. In the present case, because the patient did not have any specific medical history such as diabetes or cerebral infarction and did not take medication that might have resulted in autonomic disorders, delayed gastric emptying due to truncal vagotomy by esophagectomy was probably the major cause for bezoar formation.

Intestinal obstruction is the most prevalent complication of bezoar formation [1]. In one report, CT scans were useful to accurately diagnose intestinal obstruction caused by bezoars in more than 70% of the cases [6]. The characteristic “well-defined mass with a mottled gas pattern” is the typical finding of a bezoar, as seen on CT [7]. In our case, a structure resembling a bezoar was not identified on CT scans (Fig. 1B); thus, an accurate diagnosis was difficult. Therefore, the endoscopic study played an important role. In addition, ulcers are also frequent complications of bezoar formation due to compression on the mucosa or mechanical stimulation [8]. In our case, an ulcer was located at the same location as the bezoar (Fig. 4). Only two cases of bezoars in the reconstructed gastric conduits after esophagectomy for esophageal cancer have been reported. In one case, the patient had a hemorrhagic gastric ulcer caused by bezoars [4]. In the other case, the patient had cancer of the gastric conduit [4]. When endoscopic studies, even those that are routine follow-up studies, reveal bezoars in the reconstructed gastric conduit, it is important to consider the presence of hidden conditions such as ulcers or cancer.

The first choice of treatment for bezoars is removal by endoscopy or a surgical procedure [1]. In our case, however, a surgical procedure might have been difficult because the bezoar was located in the reconstructed gastric conduit through the posterior sternum. Recently, chemical dissolution with Coca-Cola (The Coca-Cola Company, Atlanta, GA) was reported to be an effective procedure in 50% of all patients with gastric bezoars. Moreover, a procedure with endoscopic methods such as disintegration and removal has successfully resolved more than 90% of all bezoars [9]. Although the dose of Coca-Cola has not been established, in some reports, approximately 3 L of Coca-Cola was found to be required [10, 11]. In our case, it was difficult to administer a dose that was large enough to dissolve the bezoar because of the low capacity of the reconstructed gastric conduit because of the obstruction and the possibility of vomiting and aspiration. Therefore, enzymatic dissolution followed by endoscopic removal was performed. In addition, migration of bezoars causing an ileus has been reported in patients in whom bezoars were treated with only dissolution. Disrupted fragments of bezoars are retained in the small intestine, accumulate, and grow in size, resulting in intestinal obstruction. Thus, the possibility of migrated bezoars leading to a secondary ileus should be considered when dissolution without removal is performed [12, 13].

In theory, bezoar formation might be prevented by alleviating delayed gastric emptying. Pylorus drainage procedures, such as pylorotomy and pyloroplasty, are routinely performed in many centers to prevent delayed gastric emptying due to truncal vagotomy, but the efficacy of these procedures remains controversial. [14]

On the other hand, Boshier et al. recently demonstrated the safety and efficacy of an intraoperative pyloric stretch procedure for the prevention of delayed gastric emptying following esophagectomy with reconstruction of the gastric conduit. This procedure was performed by passing a Rampley sponge holding forceps through a gastrostomy that was created proximal to the intended gastric resection margin. [15, 16] Accordingly, this intraoperative pyloric stretch procedure should be considered to prevent bezoar formation in the gastric conduit as a result of delayed gastric emptying.

To conclude, considering the possibility that bezoars can cause reconstructed gastric conduit obstruction, this case report highlights the importance of endoscopy-based diagnosis in cases wherein discharge of materials from the gastric conduit is delayed and considering obstruction due to the bezoar as a differential diagnosis in such cases.

## Data Availability

All data generated or analyzed during this study are included in this article.

## Abbreviations

CT: Computed tomography
